# SEAOP: a statistical ensemble approach for outlier detection in quantitative proteomics data

**DOI:** 10.1093/bib/bbae129

**Published:** 2024-03-31

**Authors:** Jinze Huang, Yang Zhao, Bo Meng, Ao Lu, Yaoguang Wei, Lianhua Dong, Xiang Fang, Dong An, Xinhua Dai

**Affiliations:** College of Information and Electrical Engineering, China Agricultural University, Beijing, 100083, China; Technology Innovation Center of Mass Spectrometry for State Market Regulation, Center for Advanced Measurement Science, National Institute of Metrology, Beijing 100029, China; Technology Innovation Center of Mass Spectrometry for State Market Regulation, Center for Advanced Measurement Science, National Institute of Metrology, Beijing 100029, China; College of Information and Electrical Engineering, China Agricultural University, Beijing, 100083, China; College of Information and Electrical Engineering, China Agricultural University, Beijing, 100083, China; Technology Innovation Center of Mass Spectrometry for State Market Regulation, Center for Advanced Measurement Science, National Institute of Metrology, Beijing 100029, China; Technology Innovation Center of Mass Spectrometry for State Market Regulation, Center for Advanced Measurement Science, National Institute of Metrology, Beijing 100029, China; College of Information and Electrical Engineering, China Agricultural University, Beijing, 100083, China; Technology Innovation Center of Mass Spectrometry for State Market Regulation, Center for Advanced Measurement Science, National Institute of Metrology, Beijing 100029, China

**Keywords:** outlier detection, Python, proteomics, quality control, ensemble

## Abstract

Quality control in quantitative proteomics is a persistent challenge, particularly in identifying and managing outliers. Unsupervised learning models, which rely on data structure rather than predefined labels, offer potential solutions. However, without clear labels, their effectiveness might be compromised. Single models are susceptible to the randomness of parameters and initialization, which can result in a high rate of false positives. Ensemble models, on the other hand, have shown capabilities in effectively mitigating the impacts of such randomness and assisting in accurately detecting true outliers. Therefore, we introduced SEAOP, a Python toolbox that utilizes an ensemble mechanism by integrating multi-round data management and a statistics-based decision pipeline with multiple models. Specifically, SEAOP uses multi-round resampling to create diverse sub-data spaces and employs outlier detection methods to identify candidate outliers in each space. Candidates are then aggregated as confirmed outliers via a chi-square test, adhering to a 95% confidence level, to ensure the precision of the unsupervised approaches. Additionally, SEAOP introduces a visualization strategy, specifically designed to intuitively and effectively display the distribution of both outlier and non-outlier samples. Optimal hyperparameter models of SEAOP for outlier detection were identified by using a gradient-simulated standard dataset and Mann–Kendall trend test. The performance of the SEAOP toolbox was evaluated using three experimental datasets, confirming its reliability and accuracy in handling quantitative proteomics.

## INTRODUCTION

Proteomics, significantly advanced by mass spectrometry (MS) and bioinformatics innovations, has emerged as a foundational technique in modern biological research [1–3]. The growing importance of proteomics, particularly in clinical applications, underscores the need for consistent, replicable and high-quality measurements in various laboratories and with different instruments [4, 5]. In response, a wide range of quality control (QC) tools specifically designed for proteomics experiments have been developed. These tools predominantly focus on monitoring the performance of Liquid Chromatography-Mass Spectrometry (LC-MS) systems, an essential aspect in proteomics research [6].

Historically, the National Institute of Standards and Technology (NIST) pioneered the initiative with their Perl-based program, NIST-MSQC, offering a robust set of 46 QC metrics [7]. Building upon this foundation, QuaMeter was subsequently launched to enhance its utility and address the limitations identified in MSQC [8]. This adaptive approach aimed to facilitate the broader adoption of QC tools within proteomics laboratories. Over time, additional tools like SIMPATIQCO, Metriculator, QCloud2 and QC-ART have further enriched the field by introducing web-based interfaces, interactive plots and features for comparing metrics [9–13]. In a more recent development, Zheng *et al*. introduced the ‘Chinese Quartet’ as a new proteome standard, consisting of four standard samples derived from different biological members of the same family [4, 5]. This was complemented by the launch of the Quartet Data Portal, specifically designed for proteomics QC [14]. These innovations have empowered instrument operators in their QC monitoring efforts.

These tools collectively provide diverse methods to evaluate raw data quality, analyzing precursor and fragment ion scans, chromatographic peak intensities and charge states. Some even incorporate peptide identification methods to assess factors like the rate of incomplete cleavage and the total number of peptide identifications. However, their potential is not without limitations. Some suffer from inconsistent maintenance, restricted interfaces (e.g. command-line only) or yielding merely text-based outputs. Some are excessively specialized, making assessment challenging for non-experts. Furthermore, specific tools, like MSQC, are exclusively designed to compare performances among predefined QC samples, making them unsuitable for evaluating samples of biological interest [8]. Such limitations considerably hinder the versatility and widespread adoption of QC tools, emphasizing the need for continuous improvement and user-friendly refinements.

Recent advancements in proteomic data processing software, such as MaxQuant and Spectronaut, have greatly facilitated proteomic studies [15, 16]. This progression has not only enabled effortless data acquisition, analysis and interpretation but also introduced challenges in distinguishing between high- and low-quality data and pinpointing outlier samples. In response to this challenge, several tools have been developed, each focusing on QC in quantitative proteomics. PTPQC, an R-based QC tool designed for MaxQuant results, covers 24 QC metrics in categories such as ‘ProteinGroups’, ‘Evidence’, ‘Msms’ and ‘MsmsScans’ [17]. pmartR detects potential outliers by merging exploratory data analysis (EDA) with the robust Mahalanobis distance (rMd) filter, considering factors like correlations, missing value proportions, median absolute deviation (MAD) and skewness [18]. POMAShiny uses EDA outcomes and Euclidean distances to detect outlier samples [19]. Meanwhile, the MaCProQC tool conducts QC through data completeness, efficiency of normalization and replicate similarity assessments [20]. Despite these advancements, existing methods still fall short in monitoring low-quality (outlier) data and instrument status. This gap highlights the need for a more comprehensive and robust evaluation approach in the field of proteomics QC.

To address this issue, we developed SEAOP, a Python toolkit designed for robust outlier detection in quantitative proteomics, harnessing statistical resampling ensemble approaches. Central to its design are three strategies: a multi-round resampling strategy for bias reduction, an optimized model selection scheme for identifying the most effective combination from 10 model options and statistical ensemble learning for accurate decision-making. Within the resampling strategy, samples are iteratively chosen across various cycles to mitigate bias while optimizing data representation. During the model selection phase, SEAOP incorporates ten distinct models for outlier detection, offering a total of 170 hyperparameter models. By analyzing high-quality gradient simulated data, we identified the most efficient model and hyperparameter combination. This combination is then applied in each sampling iteration for outlier evaluation. Finally, the statistical ensemble learning approach is employed, in which any samples identified as outliers are systematically counted and analyzed. This process involves using a chi-square test on the collective results from all iterations, which helps in establishing a well-defined outlier decision boundary and in selecting outliers that are statistically significant.

To demonstrate the effectiveness of SEAOP, we conducted comprehensive evaluations using three distinct quantitative proteomics datasets. Our results underscore the remarkable capability of the SEAOP toolkit not only to detect outliers but also to significantly influence data analysis. Furthermore, we confirmed that SEAOP can efficiently process MaxQuant outputs. Additionally, SEAOP offers interactive visualization tools, aiding users in clearing interpreting of the results. Overall, SEAOP stands out as a comprehensive solution for proteomics data processing, offering unmatched outlier detection, versatile data handling, real-time monitoring capabilities and user-friendly visualization features.

## MATERIALS AND METHODS

### Data collection and preparation

To evaluate the effectiveness and applicability of SEAOP, we used three large-scale datasets from label-free proteomics analysis via data-dependent acquisition MS (DDA-MS).

The first dataset was produced by our laboratory using HeLa cells. The HeLa cells, sourced from the Cell Bank of the Chinese Academy of Sciences, were processed to generate standardized reference peptide mixtures for constant LC-MS/MS QC. When the cell culture density achieved 1 × 10^^7^ cells/ml，proteins were extracted from the cells across five batches (totaling 50 culture dishes) and subsequently pooled for consistent lysate profiling. We utilized the filter-aided sample preparation (FASP) method for protein digestion, as outlined in our previous work [21]. Post-digestion, peptides were combined to produce a pooled reference peptide mixture, which was standardized and used for MS quality assessments. The peptide concentration was determined using a Multiskan Sky spectrophotometer (Thermo Fisher) employing the Bradford assay.

For the acquisition of high-quality quantitative proteomics data, peptide samples were analyzed using the Orbitrap Fusion Lumos mass spectrometer (Thermo Fisher) coupled with the Easy-nLC 1200 system. To maintain consistent data quality, each assay incorporated a 1 μg sample of the peptide mixture, undergoing a 78 min gradient separation. Specifics of the gradient and MS parameters can be found in our earlier publication [22]. Over a span of 8 months, our team successfully generated a total of 308 raw MS files for QC in HeLa proteomics analysis. Notably, four of these samples were intentionally acquired during periods when the instrument exhibited sub-optimal performance. Any data deemed of low-quality were annotated with precision by expert instrument operators. The quantitative proteomics data were obtained using the MaxQuant software (version 2.0.3.0) searching against the Uniprot/Swiss-Prot protein database [23] and used for SEAOP toolkit evaluation. The results of the protein qualitative and quantitative matrix obtained from MaxQuant can be found in Table S1.

Two additional validation datasets were acquired from the Chinese Human Proteome Project (CNHPP), which were used to characterize hepatocellular carcinoma (HCC) and lung adenocarcinoma (LUAD) [21, 24]. Both datasets, produced using the FASP and DDA-MS methods, underwent fractioning: 6 for HCC and 10 for LUAD. The HCC dataset yielded 618 raw MS files from 103 patients, while the LUAD dataset produced 2060 files from 103 patients. All files were analyzed using MaxQuant software. For further details on sample preparation and LC-MS/MS analysis, please refer to the associated primary publications. The raw data can be downloaded from the iProx database using the following IDs: IPX0000937000 and IPX0001804000 [25]. The results of the protein qualitative and quantitative matrix obtained from MaxQuant can be found in Tables S2 and S3.

### Overview of SEAOP methods

**Figure 1:**
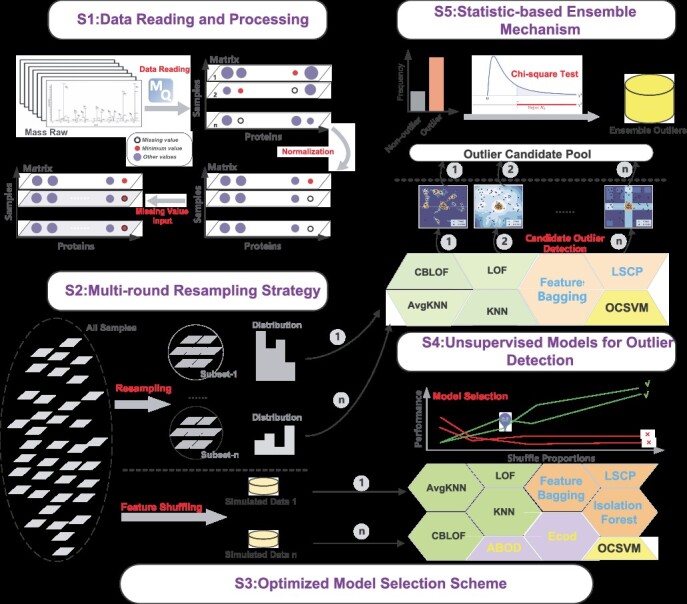
Workflow for the SEAOP toolkit. S1: Proteomics data are initially formatted into a matrix suitable for machine learning algorithm processing, following normalization and missing value input procedures. S2: Subsequently, a multi-round resampling strategy is employed to generate *n* subsets from the entire sample pool, effectively mitigating feature bias. S3: Ten machine learning models are evaluated on the 10 groups of simulated data, which are generated by manipulating the number of random feature shuffling. Then, the optimized model selection scheme is used to determine the most suitable shuffle proportions according to the model stability, and the models are filtered according to evaluation accuracy on simulated data. S4: The selected models are constructed to identify outliers based on decision boundaries learned from their respective subsets. Any samples designated as outliers are placed into the Candidate Outlier Pool. S5: The determination of whether a candidate outlier qualifies as a true outlier detection result is made by conducting a chi-square test on each candidate sample. Note: During practical application, users can directly execute S1, S2, S4 and S5 with the optimal model combinations selected in this study to obtain anomaly detection results.

SEAOP is an integrated tool purposefully designed for the detection of outlier samples in quantitative proteomics data, combining data collection, processing, evaluation and visualization functions. This tool can efficiently process outputs from maxQuant and is proficient in managing data presented in matrix format. The software included four main steps: (1) data reading and processing; (2) multi-round resampling strategy for bias reduction; (3) optimized model selection scheme for identifying the most effective combination from 10 model options; and (4) statistical ensemble learning for precise decision-making. The workflow of the toolkit is presented in Figure 1.

### Data reading and processing

The SEAOP is specifically tailored for direct integration with the protein group.txt files generated by the MaxQuant software. It efficiently processes proteomics intensity, iBAQ, LFQ data, and other matrix data, enabling an exhaustive analysis. When working with quantitative proteomics, normalization of datasets plays an important role, especially when undertaking comparative analyses. Within SEAOP, several tools are available for this purpose: (i) quantile normalization: this method standardizes sample distributions through quantile alignment, replacing each data point with the mean of its corresponding quantile. This process ensures uniform feature expression across different samples and has been validated for its effectiveness in proteomics studies, making it our chosen default normalization approach [21, 24, 26]. (ii) LOESS normalization: in proteomics, this method adjusts non-linear biases in data using localized regressions, ensuring accurate representation of protein expression variations across samples. It is also widely employed in normalization studies within the field of proteomics [20]. (iii) StandardScaler: designed to process data such that it conforms to a standard normal distribution. By using the mean and variance, it ensures that the data adhere to a normal distribution. (iv) Noscaler: as the name suggests, when this tool is selected, no scaling operations are applied to the data. These tools offer a diverse selection of options for proteomics data normalization, accommodating various needs and data characteristics.

#### Missing value imputation

Due to the biological and technical factors, such as inaccurate peak detection or values below the quantification limit, some features in proteomics samples may not be identifiable or quantifiable. To address this issue, SEAOP initially introduced an optional method for handling missing values, allowing users to selectively remove data features with missing values exceeding a specific threshold (default is 75%) across all study samples. Subsequently, for remaining missing values, the toolbox automatically fills them with the minimum value found in the dataset, a method proven optimal for such cases [27].

### Multi-round resampling strategy

The primary purpose of this method is to evaluate the stability of identified outliers, specifically, the robustness of these potential outliers to sampling variability. The method aims to determine whether these outliers can be reliably identified when building different hypothesis space via multiple subsets sampling multiple times from the same data population. Such an assessment is crucial in determining whether these outliers genuinely exist or are merely accidental results due to single training subset. Consequently, if outliers consistently exhibit a high degree of robustness to hypothesis space variability, it further supports the notion that they are indeed genuine deviations.

In this context, resampling techniques are employed to simulate perturbations in the original data. During each resampling iteration, a certain proportion of samples (by default, 80%) can be randomly selected. This method ensures that the dataset used in each run varies slightly, introducing an element of randomness and enabling a comprehensive assessment of the model’s stability and performance under different data setting. Subsequently, the selected outlier detection model is applied to each of these perturbed datasets. As each iteration of resampling might yield slightly different outliers due to the inherent variability, it is of paramount importance to analyze the results across multiple runs. By aggregating and comparing the detected outliers in various iterations, we can obtain a more complete understanding of the consistent and genuine deviations in the data.

### Optimized model selection scheme

#### Outlier detection models

Traditional outlier detectors, such as interquartile range (IQR), *Z*-score, 3sigma and Grubbs’ test, are well recognized in many domains. However, their efficacy diminishes in the context of proteomic data due to its inherent high-dimensional characteristics. Given the limitations of these conventional models, machine learning–driven techniques emerge as more appropriate alternatives [28]. Their strength lies in effectively analyzing complex statistical patterns inherent to high-dimensional datasets, a fundamental necessity in proteomic data analysis. Within SEAOP, we have incorporated ten leading machine learning algorithms tailored for outlier analysis (Table 1), which can be broadly categorized into three groups: (1) proximity-based algorithms, including local outlier factor (LOF), *K*-nearest neighbors (KNN), average *K*-nearest neighbors (AvgKNN), one-class support vector machine (OCSVM), clustering-based local outlier factor (CBLOF), isolation forest (IForest) and angle-based outlier detection (ABOD), which exploit the distance between data points (peptides, proteins, etc.), regarding objects that are rare events away from others; (2) distribution-based algorithms, including histogram-based outlier score (HBOS), empirical-cumulative-distribution-based outlier detection (ECOD), which spots outliers via fitting the distribution of a variable; and (3) ensembling-based algorithms, including extreme boosting–based outlier detection (XGBOD), FeatureBagging (FeatB) and locally selective combination in parallel (LSCP), which ensemble models to pursue better accuracy.

**Table 1:** Unsupervised outlier detection algorithms included in SEAOP

Methods	Type	Year	References	Authors	Model counts
LOF	Proximity	2000	LOF: identifying density-based local outliers	Breuniq *et al*., 2000 [29]	15
KNN	Proximity	2000	Efficient algorithms for mining outliers from large data sets	Ramaswamy *et al*., 2000 [30]	30
AvgKNN	Proximity	2000	August. Fast outlier detection in high-dimensional spaces	Angiulli & Pizzuti, 2002 [31]	30
CBLOF	Proximity	2003	Discovering cluster-based local outliers	He *et al*., 2003 [32]	15
OCSVM	Linear Model	2001	Estimating the support of a high-dimensional distribution	Schölkopf *et al*., 2001 [33]	15
ABOD	Probabilistic	2008	Angle-based outlier detection in high-dimensional data	Kriegel *et al*., 2008 [34]	15
ECOD	Probabilistic	2022	ECOD: Unsupervised Outlier Detection Using Empirical Cumulative Distribution Functions	Li *et al*., 2022 [35]	5
IForest	Ensembles	2008	Isolation forest	Liu *et al*., 2008 [36]	15
FeatB	Ensembling	2005	FeatureBagging for outlier detection	Lazarevic & Kumar, 2005 [37]	15
LSCP	Ensembling	2019	LSCP: locally selective combination in parallel outlier ensembles	Zhao *et al*., 2019 [38]	15

It is worth noting that, in the field of machine learning, factors influencing model outcomes extend beyond the type of the model itself, equally central to model performance are its hyperparameters. The settings of these hyperparameters, typically tailored to the specific problem and dataset at hand, markedly affect the efficacy and generalization capabilities of the employed statistical learning models. In the context of proteomics data evaluation, it typically lacks known labels. To address challenges posed by the lack of supervision and reduce their impact on final results, within the SEAOP toolkit, we have specifically designed multiple hyperparameter types for all our unsupervised models. These key hyperparameters included parameters such as the number of iterations (for LOF, KNN, CBLOF, ABOD and IForest), random initialization values (for LSCP and FeatureBagging) and distinct metrics for quantifying dissimilarity between different samples (for KNN, AvgKNN and OCSVM), among others. Through various model and parameter combinations, SEAOP encompasses a total of 170 hyperparameter combination models. For detailed model information, please refer to Table 1.

#### Data simulation and model selection

To determine the optimal model parameter combination for SEAOP, we utilized the HeLa dataset for data simulation and model selection. To begin, we refined the dataset by using protein identification numbers, correlation coefficients and existing outlier detection methods to eliminate potential outliers, ensuring data stability and reliability. After that, we divided the high-quality data into 10 groups, each with about 30 samples (totaling 302 samples). For each experimental iteration, one group was selected to simulate outlier features, systematically adjusting feature data in 10% increments up to 100%, increasing by 10% in each step (feature shuffling). This approach allows for a systematic assessment of the robustness of different models under varying degrees of feature changes. The entire process is repeated 10 times.

For selecting the best model combination, our focus is on three key elements: stability of the model, its accuracy and its robustness. These aspects are evaluated using coefficient of variations (CV), accuracy metrics and the number of shuffled features. Essentially, a preferable model would be one that exhibits stability (indicated by lower CV values), maintains high accuracy (reflected by higher accuracy scores) and demonstrates robustness, particularly at lower shuffle proportions. To begin, we used the Mann–Kendall (MK) trend test to determine the optimal feature shuffle proportion [39], analyzing how CV values change with varying levels of feature shuffling. Stability is achieved when a model’s CV values do not significantly fluctuate with different shuffle proportions. A voting mechanism is then employed to identify the feature shuffle proportion where most models attain stability. We select models as candidates if they maintain an accuracy of over 90% at or below this optimal shuffle proportion. This threshold is set at 90% rather than 100% to allow for fault tolerance, acknowledging the difficulty in ensuring that simulated data are completely true and enhancing the models’ overall robustness by incorporating fault tolerance.

### Statistic-based ensemble mechanism

Ensemble learning integrates the salient features of two or more models to achieve a consensus in prediction. This method of integration offers enhanced robustness compared to individual models, primarily because it mitigates the variability in prediction errors. Among various strategies documented in the literature, the majority voting system, which aggregates decisions from a collective of models, is recognized as one of the most foundational and fundamental methods. Nonetheless, this approach can introduce notable inconsistencies when the number of votes is nearly equivalent among competing outcomes. To address this challenge, chi-square statistical analysis has been incorporated into the voting phase. In this strategy, all samples are divided into two distinct types: normal and outliers. Given that the chi-square test does not explicitly indicate the nature or strength of the relationship, samples primarily identified as normal are excluded. For each potential outlier, a chi-square test is conducted, marking it as an outlier at a 95% confidence level.

### Tool implementation

All functions in SEAOP were compiled in Python (Version 2 and 3 using six, numpy, scipy and scikit-learn, PyOD), and it was deployed on a server with Intel (R) Xeon (R) CPU E5-2683 running on the Ubuntu release 16.04.12, operating system. It not only perfectly inherits several key advantages of PyOD, such as just-in-time (JIT) compilation, compatibility across major operating systems, a unified application programming interface (API), detailed documentation and interactive examples, but it also provides a multiprocessing approach. In the development environment described in this document, the multiprocessing approach has been shown to significantly improve runtime speeds by more than 30 times. In addition, the source codes of SEAOP are available on the GitHub repository: https://github.com/whisperH/SEAOP under the massachusetts institute of technology (MIT) license. The detailed installation and operation manual can be found in supplementary notes on GitHub repository.

### Statistical analysis

Outlier samples in terms of protein identification numbers and correlation coefficients were identified through Grubbs’ test. To detect differentially expressed proteins within the HCC and LUAD datasets, a straightforward linear model combined with moderated *t*-statistics was utilized [21]. This analysis was performed using the R/Bioconductor package, limma v.3.24.15. Additionally, for the HCC and LUAD clustering analysis, the Euclidean distance and average clustering algorithm were employed.

## RESULTS

### Basic description of datasets

#### HeLa data

**Figure 2:**
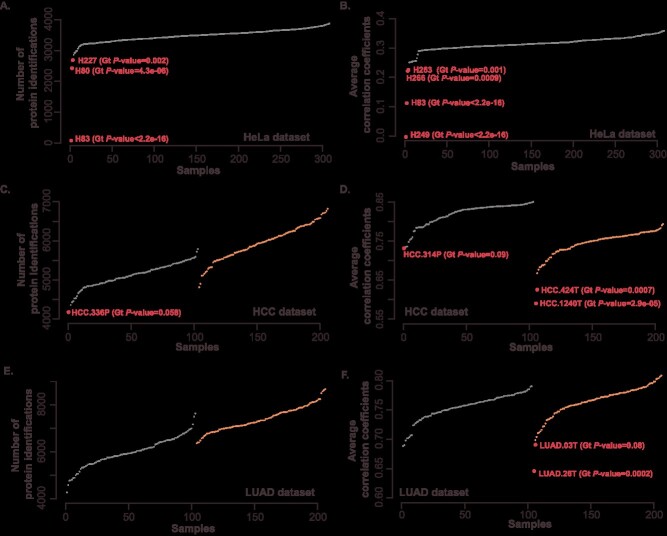
Outlier detection in the HeLa, HCC and LUAD datasets. (**A**, **B**) Detection of outliers in the HeLa datasets. Outliers were identified using Grubbs’test based on either the number of protein identifications (A) or the correlation coefficients (B). To ensure clarity, these outliers are specifically highlighted through textual annotations. (**C**–**F**) Detection of outliers in the HCC and LUAD datasets. The identification of outliers was also determined using Grubbs’test, based on either the number of protein identifications or the correlation coefficients. The NAT samples are positioned on the left, tumor samples on the right, with outliers specifically marked through textual annotations. It should be noted that NAT and tumor samples were calculated separately.

We intermittently collected MS data from 308 HeLa cell samples on the same mass spectrometer and identified a total of 6451 proteins. Protein identification levels in MS can reflect sample quality and instrument performance, with better conditions leading to higher accuracy in identification. Using Grubbs test, we conducted a statistical examination on the protein identification numbers of 308 samples, identifying three outlier samples (numbered H80, H83 and H227, *P*-value <0.05), with protein identification numbers of 2425, 75 and 2689, respectively (Figure 2A). After removing these three outlier samples, the range of protein identification numbers for the remaining samples was 2863–3878 (Table S4).

Furthermore, through correlation analysis, we discovered that samples H83, H249, H263 and H266 exhibited significantly lower correlation compared to the others (Grubbs test, *P*-value <0.05, Figure 2B), categorizing them as outliers as well (Table S4). Consequently, a total of six potential outlier samples (H80, H83, H227, H249, H263 and H266) were identified in the HeLa dataset. Additionally, the outliers detected by the MaCProQC tool corroborated the outlier status of H80, H83 and H227.

#### HCC and LUAD data

In both HCC and LUAD datasets, a total of 103 paired normal adjacent tissues (NATs) and tumor samples were analyzed. Although no outlier samples were detected in either dataset through Grubbs’ test, correlation clustering analysis revealed suspected outliers in each dataset. In the HCC dataset, NAT sample HCC.336P (*P*-value = 0.06) and HCC.314P (*P*-value = 0.09) and tumor samples 424T and 1240T (*P*-value <0.01) were flagged as candidate outliers (Table S4 and Figure 2C and D). While, within the LUAD data, tumor samples LUAD.26T (*P*-value <0.01) and LUAD.03T (*P*-value = 0.08), were characterized as suspected outlier samples (Table S4 and Figure 2E and F). However, the identification of outlier samples through correlation coefficients paired with unsupervised clustering methods exhibits a level of subjectivity and fails to achieve clear discriminative criteria statistically. These findings highlight the potential differences in identifying outlier samples when employing distinct statistical analysis methods, underscoring the importance of developing a robust method for outlier sample detection. The employment of the SEAOP toolkit emerges as a proficient approach towards addressing this issue.

### SEAOP model evaluation and selection

In our SEAOP framework, 10 diverse outlier detection algorithms were integrated, comprising 170 distinct parameter models. The objective was to identify the most effective hyperparameter model combinations for SEAOP toolbox on simulated data. We selected the HeLa dataset, known for its consistent condition-derived data, as the benchmark. Before analysis, six potential outliers (H83, H249, H263, H266, H80, H227) identified through statistical analysis were removed, leaving us with 302 samples for constructing the simulated dataset (Materials and methods). These samples were divided into 10 groups, each containing approximately 30 samples. In each test iteration, one group was selected, and outlier samples were simulated by ‘feature shuffling’—incrementally altering feature data by 10% increments, from 10% to 100%. This process was repeated 10 times.

**Figure 3:**
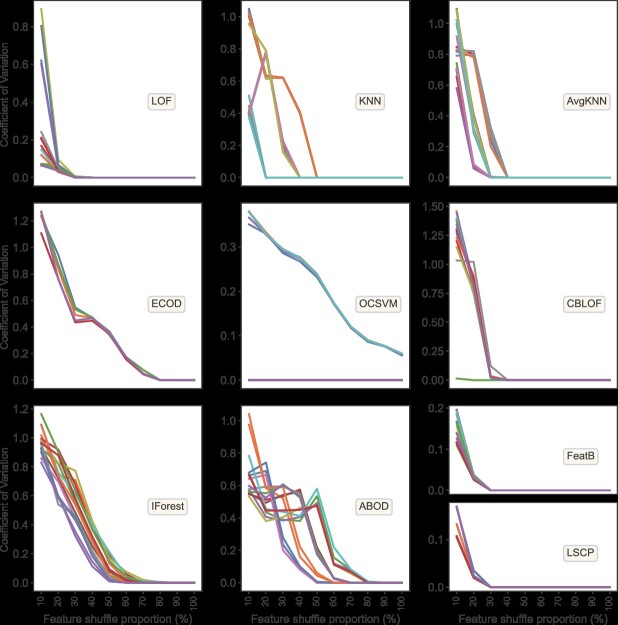
The relationship between coefficient of variation (CV) values and feature shuffle proportions. The analysis includes results from 170 hyperparameter models across 10 algorithms. The *x*-axis represents the gradation of feature shuffling proportions, while the *y*-axis indicates the CV values, calculated from the outlier detection accuracies of 100 tests (10 groups × 10 repeats) for each single model at different shuffle proportion.

We first evaluated the impact of the feature shuffle proportion on model stability in simulations, by monitoring variations in the CV values at different shuffle proportions. As illustrated in Figure 3, all test models displayed a steady decline in the CV values of accuracy as the shuffle proportions increased. This indicated better precision and stability in identifying outliers when feature changes were more significant. Notably, the FeatB, LSCP and LOF models showed minimal variation in CV values with 10% and 20% feature changes. At 30% feature change, their CV values almost reached zero, reflecting high stability in outlier detection.

Moreover, models using the KNN, AvgKNN and CBLOF algorithms exhibited higher CV values at lower shuffle proportions (10% and 20%). However, as the feature shuffle proportion increased to 30%, models with different hyperparameters exhibited distinct trends in CV values, with some notably converging toward zero (Figure 3). Meanwhile, models like ECOD, IForest and ABOD showed diminishing CV values as the feature shuffle proportion increased, only stabilizing in accurate outlier detection when the proportion exceeded 60%, as indicated by CV values nearing zero. Additionally, for the OCSVM algorithm, significant differences in CV values were observed with different model hyperparameters, particularly the ‘rbf’ and ‘sigmoid’ kernels, which exhibited consistent performance in handling outlier detection. This pattern suggests that specific combinations of hyperparameters in these algorithms are exceptionally proficient at detecting outlier samples.

The proportion of feature shuffling plays a crucial role in determining both the quantity and quality of the models selected. Setting this proportion too low may result in the exclusion of a significant number of models, thereby increasing the likelihood of overfitting. This could impair the model’s efficacy on diverse real-world datasets and diminish its capability to accurately identify outliers. Conversely, excessively high feature shuffle proportions could lead to the retention of under-fitted models, adversely impacting the overall accuracy of the outcomes.

To determine the optimal feature shuffle proportion for each of the 170 models, we divided the range of feature shuffle proportions into nine segments (e.g. Seq1 covering 10–100%, up to Seq9 from 90% to 100%). The MK trend test was then applied to identify the most suitable segment for each model. The goal was to find the longest segment that maintained a 95% confidence level in terms of statistical significance. The starting point of this longest segment was designated as the ‘detection point’. However, merely identifying the ‘detection point’ was not sufficient. To ensure the practical utility of the models, an additional criterion was applied: only models exhibiting an outlier detection accuracy above 90% at their respective ‘detection points’ were considered viable. Models failing to meet this threshold were excluded. The distribution of the selected hyperparameter models across different ‘detection points’ (referring to feature shuffle proportions) is illustrated in Table 2.

**Table 2:** Distribution of selected models across different feature shuffle proportions. Shuffle proportions refer to the percentage of altered features relative to the total number of features

Shuffle ratios	10%	20%	30%	40%	50%	60%	70%	80%	90%
Model counts	6	49	36	6	2	2	2	0	0
Model selected	Yes	Yes	Yes	No	No	No	No	No	No

**Figure 4:**
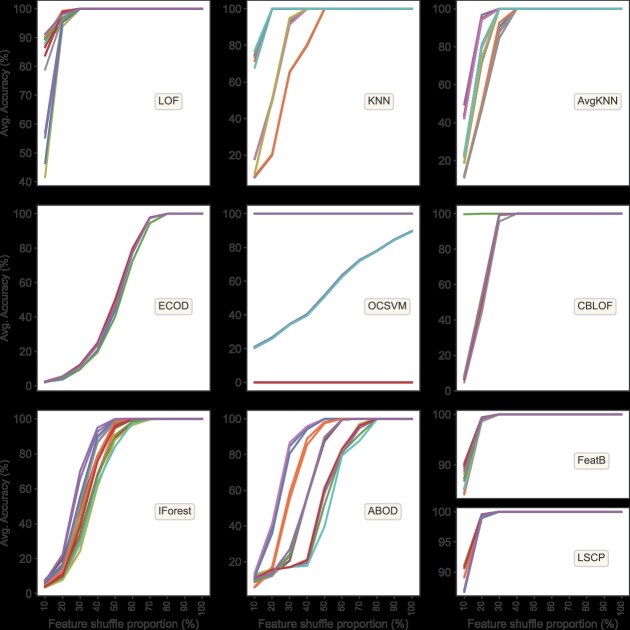
The relationship between mean accuracy and feature shuffle proportions. The analysis includes results from 170 hyperparameter models across 10 algorithms. The *x*-axis represents the gradation of feature shuffling proportions, while the *y*-axis shows the mean accuracy, obtained from 100 tests (10 groups × 10 repeats) for each single model at different shuffle proportion.

To achieve a balance between the precision of the training model and its generalization capacity, a collective decision-making process utilizing a voting method was implemented. This resulted in the choice of a 20% feature shuffle proportion as the optimal balance point. To further ensure the stability of CV values, the node subsequent to this balance point was selected as the definitive criterion for model selection, with a threshold set at 30%. Models exhibiting robust performance with feature shuffle proportions below 30% were selected for the development of SEAOP’ integrated outlier detection model (Figure 4). Following this strategy, SEAOP narrowed down its selection from an original pool of 170 hyperparameter models to 91, covering seven distinct outlier detection algorithms. The selected models include LOF, KNN, AvgKNN, OCSVM, SBLOF, FeaB and LSCP, as detailed in Table 3.

**Table 3:** Comparison of hyperparameter models pre- and post-model selection in the SEAOP framework

Outlier detection algorithms	Models (before)	Models (after)	Outlier detection algorithms	Models (before)	Models (after)
LOF	15	15	CBLOF	15	15
KNN	30	10	IForest	15	0
AvgKNN	30	16	ABOD	15	0
ECOD	5	0	FeatB	15	15
OVSCM	15	5	LSCP	15	15
Total	/			170	91

### Assessment of SEAOP in outlier detection across three datasets

**Figure 5:**
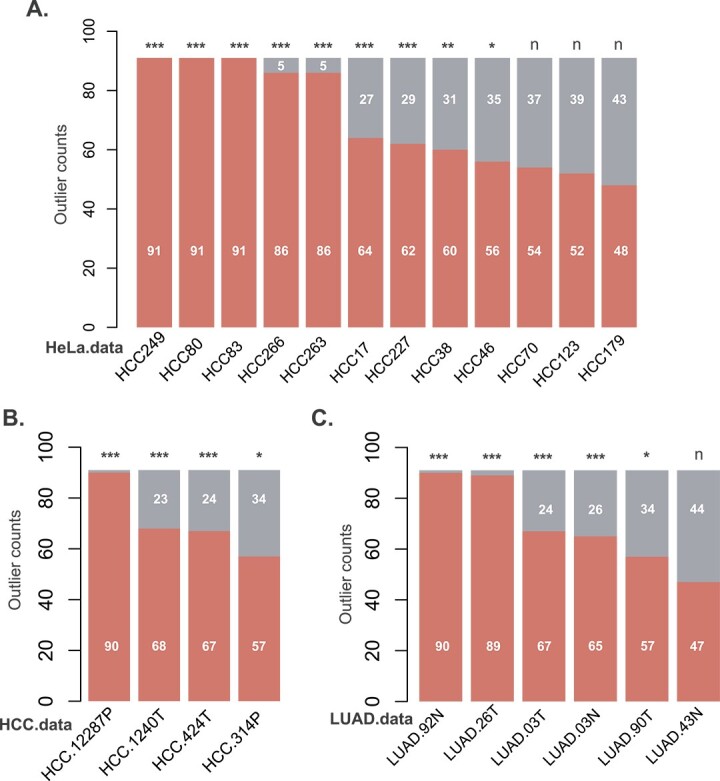
Results of SEAOP for (**A**) HeLa, (**B**) HCC and (**C**) LUAD datasets. Significance levels are denoted by asterisks: ^***^ indicates *P*-value <0.001, ^**^ indicates *P*-value <0.01 and ^*^ indicates *P*-value <0.05 under the chi-square test condition.

Next, the SEAOP toolbox was applied to HeLa, HCC and LUAD datasets to evaluate its capability in identifying outliers. In the HeLa dataset, 12 samples were initially identified as potential outliers, including H17, H38, H46, H70, H80, H83, H123, H179, H227, H249, H263 and H266 (Figure 5A). A detailed analysis revealed that samples H70, H123 and H179 had classification frequencies as outliers or non-outliers of 54/37, 52/39 and 48/43, respectively. Chi-square test outcomes indicated *P*-values of 0.075 for H70, 0.173 for H123 and 0.600 for H179, suggesting these samples should not be consider as outliers. Overall, through the SEAOP analysis, a total of nine samples were ultimately identified as outliers in the HeLa dataset, specifically H17, H38, H46, H80, H83, H227, H249, H263 and H266, all with *P*-values below 0.05 (Table S5). This group includes all the six outlier samples identified through statistical testing in the basic data description section.

In the HCC and LUAD datasets, we categorized the samples into two main groups based on two distinct labels: NATs (non-tumor-adjacent tissues) and tumors. Following this, the SEAOP method was utilized to identify the outlier samples within each category. As illustrated in Figure 5B, in the HCC datasets, we identified a total of four outlier samples, which include NAT samples HCC.314P and HCC.12287P, as well as tumor samples HCC.424 T and HCC.1240 T (*P*-value <0.001, Table S5). Remarkably, three of these were previously identified by Grubbs’ test and the associated correlation coefficient results (Figure 2C and D). On the other hand, in the LUAD dataset, we were able to identify a total of five outlier samples, encompassing NAT samples LUAD.03N (*P*-value = 0.019), and LUAD.92N and tumor samples LUAD.03 T, LUAD.26 T and LUAD.90 T (*P*-value <0.001, Figure 5C and Table S5). Notably, two of these were previously identified by Grubbs’ test and the associated correlation coefficient results (Figure 2F).

Collectively, these findings suggest that the SEAOP method possesses enhanced accuracy in identifying outlier samples. It not only successfully identifies outliers in diverse datasets but also corroborates findings from traditional methods like the Grubbs’ test, demonstrating its robustness and reliability in outlier detection across various sample types.

### Outlier detection: SEAOP versus single-model methods

Next, we conducted a comparative analysis to assess the performance in detecting outlier samples between the SEAOP toolbox and other single-model approaches, with a focus on the HeLa, HCC and LUAD datasets. Within the HeLa datasets, SEAOP excelled, displaying unparalleled consistency and accuracy in outlier detection compared to other well-established methodologies (Table 4). Notably, SEAOP identified all potential outlier samples detected by any model, highlighting its robust detection capabilities. In contrast, single-model methods such as data completeness, efficiency of normalization and replicate similarity—all components of the MaCProQC tool—exhibited a markedly lower efficacy, identifying outliers in only a handful of samples. While intermediate methods, including Grubbs’ test, CBLOF, AvgKNN, KNN, OCSVM and LOF, exhibited a moderate level of detection, identifying outliers in about five to six samples, they did not achieve the breadth of detection observed with SEAOP. FeatB, although highly effective with detections in eight samples, did not reach SEAOP’s comprehensive detection rate. LSCP was noteworthy, identifying all outliers similar to SEAOP and affirming its viability as an alternative for outlier detection.

**Table 4:** Comparative analysis of outlier detection performance in HeLa dataset: SEAOP Toolbox versus single-model approaches

Outlier samples	H17	H38	H46	H80	H83	H227	H249	H263	H266
SEAOP (ours)	√	√	√	√	√	√	√	√	√
Data completeness	–	–	–	√	√	√	–	–	–
Replicate similarity	√	–	–	√	√	–	√	√	√
Efficiency of normalization	–	–	–	√	√	–	–	–	–
Grubbs’ test	–	–	–	√	√	√	√	√	√
LSCP	√	√	√	√	√	√	√	√	√
CBLOF	–	–	–	√	√	–	√	√	√
FeatureBagging	√	√	–	√	√	√	√	√	√
KNN	–	–	√	√	√	–	√	√	√
AvgKNN	√	–	–	√	√	–	√	√	√
LOF	–	–	–	√	√	√	√	√	√
OCSVM	√	√	–	√	√	√	√	–	–

In the HCC and LUAD datasets, the MaCProQC tool’s methods failed to recognize potential outlier samples, suggesting inherent limitations in these methods for practical applications (Tables 5 and 6). Within the HCC dataset, methods like CBLOF and OCSVM typically identified only a single outlier sample. However, FeatB and LSCP maintained robustness, matching the number of outlier detections by SEAOP, with the LOF algorithm also performing significantly well by identifying all outliers. Within the HCC dataset, methods like CBLOF and OCSVM typically identified only a single outlier sample (Table 5). In the LUAD dataset, most machine learning algorithms performed admirably, detecting three to four valid outlier samples, indicating a certain degree of stability in outlier detection by these models. Interestingly, it was observed that LSCP failed to recognize some outliers in this dataset (Table 6). These findings indicate that while some algorithms demonstrate effectiveness in outlier detection, there are variations in their performance across different datasets.

**Table 5:** Comparative analysis of outlier detection performance in HCC dataset: SEAOP Toolbox versus single-model approaches

Outlier samples	HCC424T	HCC1240T	HCC1228P	HCC.314P
SEAOP (ours)	√	√	√	√
Data completeness	–	–	–	–
Replicate similarity	–	–	–	–
Efficiency of normalization	–	–	–	–
Grubbs’ test	√	√	–	–
LSCP	√	√	√	√
CBLOF	–	–	–	√
FeatureBagging	√	√	√	√
KNN	√	√	–	√
AvgKNN	–	–	√	–
LOF	√	√	√	√
OCSVM	–	–	√	–

**Table 6:** Comparative analysis of outlier detection performance in LUAD dataset: SEAOP Toolbox versus single-model approaches

Outlier samples	LUAD.03 T	LUAD.26 T	LUAD.90 T	LUAD.03 N	LUAD.92 N
SEAOP (ours)	√	√	√	√	√
Data completeness	–	–	–	–	–
Replicate similarity	–	–	–	–	–
Efficiency of normalization	–	–	–	–	–
Grubbs’ test	√	√	–	–	–
LSCP	√	√	–	√	√
CBLOF	-	√	–	√	√
FeatureBagging	√	√	–	√	√
KNN	√	√	√	–	√
AvgKNN	–	√	–	–	√
LOF	√	√	–	√	√
OCSVM	–	√	√	–	√

Overall, the results underscore the efficacy of the SEAOP toolbox’s ensemble approach in detecting outliers. By integrating outputs from various models, SEAOP not only enhances the precision of detection but also ensures a more extensive coverage, reducing the probability of missed detection.

### Effect of outlier removal in HCC and LUAD data analysis

**Figure 6:**
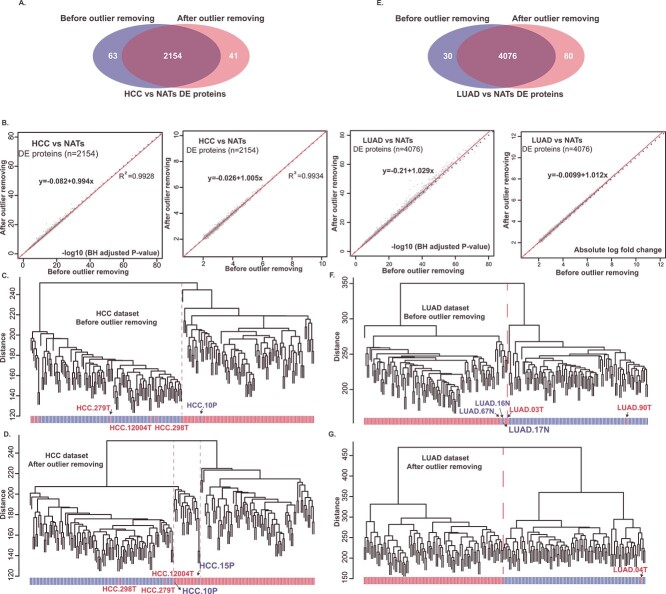
Performance evaluation of ensemble results. (**A**, **B**) Differentially expressed (DE) proteins in tumor and NAT samples, both before and after the removal of outlier samples, for HCC (A) and LUAD (B) datasets. The left ellipse represents samples with outliers， while the right side denotes samples after outlier removal. (**C**) Linear relationship results for BH-adjusted *P*-values and absolute log fold change, both before and after outlier sample removal, for HCC and LUAD datasets. (**D**–**G**) Unsupervised clustering of tumor and NAT samples in the HCC (D, E) and LUAD (F, G) datasets, before and after the exclusion of outliers. We employed the Euclidean distance and average linkage method for clustering. In the clustering results, we annotated the anomalous samples.

In our HCC datasets, we compared the differentially expressed (DE) proteins in tumor and NATs samples before and after the removal of outlier samples. Remarkably, the total count of identified DE proteins remained relatively constant: 2217 before removal and 2195 after, with an overlap of 2154 proteins (BH *P*-value <0.01 and absolute (log fold change) >2, Figure 6A). Linear relationship analysis suggested that the statistical significance remained relatively stable after outlier exclusion. Yet, there was a minor increase in the significance of fold-change, modeled by the equation *y* = −0.026 + 1.005*x* (Figure 6B). The slight elevation in the intercept (1.005) was not markedly evident. Using unsupervised clustering, the differential proteomic profiles, before outlier exclusion, failed to clearly discriminate between HCC tumor and NAT samples, as evidenced by a significant overlap of eight tumor samples with NAT samples (Figure 6C). However, after outlier exclusion, the discrimination improved with fewer overlapping samples (Figure 6D). Notably, a group of HCC samples exhibited a strong correlation with NAT samples. This finding consisted of a subgroup we previously reported in the literature that retains liver functions.

In the LUAD datasets, the number of DE proteins identified between tumor and NAT samples increased from 4106 (before) to 4156 (after) proteins (BH adjusted *P*-value <0.01 and absolute (log fold change) > 2, Figure 6E). The number of repeatedly identified DE proteins was 4076. Through linear relationship analysis, we found that the exclusion of outlier samples significantly increased the statistical significance and fold change of the identified differential proteins. This is reflected in the model parameters *y* = −0.0099 + 1.012*x* (*P*-value) and *y* = −0.21 + 1.029*x* [absolute (log fold change)], with a notable increase in the intercept (Figure 6B). Using unsupervised clustering, we observed that the removal of outlier samples enhanced the distinction between tumor and NAT samples (Figure 6F and G). These results suggest that outlier removal indeed improves and elevates the analysis outcomes of the experimental data, indicating the practical applicability and value of the SEAOP package.

### Visualization tool and decision boundaries

In the context of outlier detection, decision boundaries play a vital role in intuitively representing the distribution relationship between non-outlier and outlier samples. They serve as a critical auxiliary tool for evaluating the reliability of a model and visually highlighting the presence of outliers. Presently, most existing visualization methods for decision boundaries are limited to two-dimensional features or employ dimension reduction techniques, such as principal component analysis, to project high-dimensional features into a two-dimensional space, subsequently visualizing them using contour plots or boundary maps.

However, it’s worth noting that the exceptionally high dimensionality of protein MS data can lead to substantial information loss when attempting to reduce it to just two dimensions. This limitation can hinder models from effectively capturing the underlying distribution of the samples.

**Figure 7:**
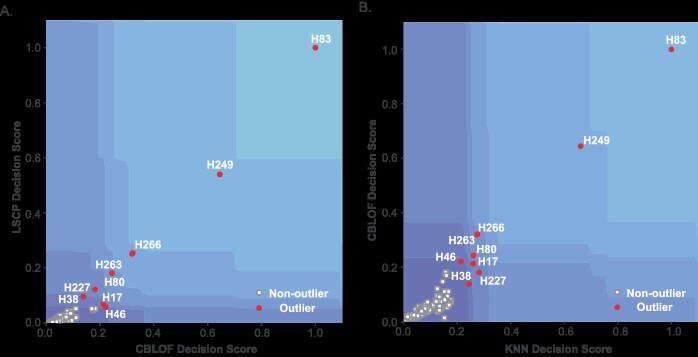
Illustration of joint decision boundaries among models. (**A**) Decision boundaries constructed by CBLOF and LSCP models; (**B**) Decision boundaries constructed by KNN and CBLOF models. The horizontal and vertical axes represent a single model’s confidence level in detecting outliers. In the figure, squares denote non-outliers, while circles denote outliers. Different color gradients depict decision boundaries reflecting various levels of confidence.

In contrast to dimension reduction algorithms, the confidence score for identifying outliers from a single model considers all features comprehensively and effectively reflects the characteristics of the sample distribution. Moreover, given the ensemble nature of our approach, the joint decision boundary can be visualized in a confidence space composed of scores from various models. Specifically, we calculate the outlier score for each sample in a single model with various hyperparameters by taking the average, with a higher score indicating a greater likelihood of the sample being classified as an outlier under that specific model, and conversely for non-outliers. Furthermore, during the visualization process, we also account for the counts of samples classified as non-outliers or outliers, with the results being presented in Figure 7.

## DISCUSSION

In this study, we have introduced SEAOP, a novel statistical ensemble-based toolkit, specifically designed for outlier detection in quantitative proteomics data. SEAOP distinguishes itself by integrating multi-sampling techniques, unsupervised outlier detection algorithms and ensemble statistical analysis, enhancing both the stability and precision of outlier detection. Its effectiveness has been validated through comprehensive testing on both simulated and experimental datasets. Remarkably, SEAOP not only identifies outlier samples using traditional statistical or single-model methods but also succeeds in detecting outliers through an extensive analysis of protein expression levels. This approach effectively overcomes the limitations inherent in traditional statistical methods, thereby ensuring greater precision and robustness.

In the SEAOP toolkit, we initially incorporated 10 unsupervised outlier detection algorithms, covering a diverse range of 170 hyperparameter models, all aimed at accurately detecting outlier data. To identify the most effective combination of these hyperparameter models, we developed a specialized methodology for evaluating unsupervised outlier detection algorithms. Our approach includes the generation of simulated data and a comprehensive assessment of model performance. Specifically, we primarily adopted the strategy of feature shuffling to simulate various types of outlier sample datasets and employed the MK trend test to assess the accuracy and stability of the different hyperparameter models across different shuffling proportions. Through a consensus voting method, we determined the optimal feature shuffling proportion, set at 30%, for use in data simulation. Ultimately, we selected seven 91 hyperparameter models across seven algorithms for SEAOP toolkit. This rigorous evaluation and selection process not only allowed us to precisely adjust the hyperparameter settings of each model but also enabled us to compare the performance of different models under various parameter configurations, ensuring the selected models demonstrate the highest stability and accuracy in data analysis, providing a powerful and reliable tool for outlier detection in proteomics.

In practical application, the SEAOP toolkit’s performance was compared with traditional single-model methods for outlier sample detection in HeLa, HCC and LUAD datasets. The results clearly demonstrated SEAOP’s advantages. In these datasets, SEAOP exhibited exceptional consistency and accuracy. Notably, in the HeLa dataset, SEAOP successfully identified all possible outlier samples. This level of performance significantly surpassed that of single-model methods like MaCProQC, which primarily focus on data completeness, efficiency of normalization and replicate similarity [20]. Intermediate methods such as Grubbs’ Test and CBLOF, as well as advanced methods like FeatB and LSCP, performed well in identifying certain outlier samples. However, they could not match SEAOP in terms of detection range and comprehensiveness. Additionally, in the HCC and LUAD datasets, traditional tools like MaCProQC failed to effectively identify outlier samples, while SEAOP showed higher stability and accuracy. The diversity and complementarity of the different hyperparameter models in SEAOP enhanced outlier sample detection. The identified outlier samples by different models showed strong complementarity, underscoring the importance of adopting multi-model ensemble methods. These results highlight the SEAOP ensemble method’s effectiveness in improving detection accuracy and coverage, thereby reducing the possibility of missed detections. This makes SEAOP a more comprehensive and reliable solution for handling high-dimensional and complex proteomics data. Overall, SEAOP’s ensemble method is demonstrably superior to traditional single-model methods, especially when dealing with complex datasets.

Furthermore, SEAOP’s multi-round resampling strategy and statistical ensemble learning method effectively reduce bias and improve the reliability of detection results when establishing different hypothesis spaces and conducting multiple samplings. This method is very important for ensuring the accuracy and reliability of research conclusions, especially in complex data environments with no clear labels.

Overall, we have pioneered an integrated statistical ensemble method for outlier detection, exhibiting notable superiority over single-model decision algorithms. Moreover, we have developed a robust visualization scheme for presenting outlier samples, ensuring a user-friendly interactive experience. Notably, in simulated datasets, we have thoroughly validated the efficacy of SEAOP, highlighting its potential as a fundamental tool for proteomics data quality assessment and control, given the provision of clean QC data. Looking ahead, we aim to further refine and broaden the capabilities of SEAOP to address a broader spectrum of challenges encountered in proteomics data analysis.

**Key Points**

- Ensuring high data quality, particularly in the management of outliers, is crucial for obtaining accurate and reliable results in omics studies.
- We introduced SEAOP, a user-friendly Python toolkit, that is designed to significantly enhance outlier detection through the integration of multi-round resampling, unsupervised outlier detectors and statistical ensemble learning, ensuring comprehensive data evaluation and accurate identification of outliers.
- We demonstrated the effectiveness of SEAOP for screening aberrant samples using quantitative proteomics data, indicating its applicability to various types of matrix sequencing data, such as transcriptomics.

## Supplementary Material

Table_S1_The_quantitative_proteomics_data_of_HeLa_samples_bbae129

Table_S2_The_quantitative_proteomics_data_of_LUAD_samples_bbae129

Table_S3_The_quantitative_proteomics_data_of_LUAD_samples_bbae129

Table_S4_The_identification_numbers_and_correlation_bbae129

Table_S5_The_outlier_samples_detected_by_SEAOP_toolbox_bbae129

## Data Availability

The raw proteomics data can be downloaded from the iProx database using the following IDs: IPX0000937000 and IPX0001804000. The results of the protein qualitative and quantitative data can be obtained from the online supplementary data section of NAR.

## Author Biographies

**Jinze Huang** is a PhD Student at the College of Information and Electrical Engineering, China Agricultural University. His main research interests are bioinformatics, pattern recognition.

**Yang Zhao** is an associate researcher at the National Institute of Metrology (NIM, China), primarily engaged in research on proteomics, bioinformatics, and computational biology.

**Bo Meng** is an assistant researcher at the National Institute of Metrology (NIM, China), mainly involved in the development of new technologies for proteomics and research on key metrological technologies.

**Ao Lu** is a graduate student at the National Institute of Metrology (NIM, China), primarily involved in completing proteomic analysis work.

**Yaoguang Wei** is a professor of artificial intelligence at China Agricultural University. His research interests are in the area of data mining and image processing.

**Lianhua Dong** is a professor and vice director for the Center of Advance Measurement of Science at National Insitute of Metrology. Her research interests are developing highly accurate methods and reliable reference materials to support the measurement in bioanalysis.

**Xiang Fang** is a leading authority in the field of mass spectrometry informatics at the National Institute of Metrology (NIM, China). His expertise encompasses the design and development of mass spectrometry instruments, advancements in single-cell technology, and the study of data models related to mass spectrometry information.

**Dong An** is a professor at the College of Information and Electrical Engineering, China Agricultural University. Her main research interests are bioinformatics, computer vision, pattern recognition.

**Xinhua Dai** received her PhD from the Institute of Chemistry Chinese Academy of Sciences. She is currently a professor at the National Institute of Metrology (NIM, China). Her research interests focus on biochemical metrology, reference materials, mass spectrometry technology and instruments.
